# Therapeutic Perspectives for Inflammation and Senescence in Osteoarthritis Using Mesenchymal Stem Cells, Mesenchymal Stem Cell-Derived Extracellular Vesicles and Senolytic Agents

**DOI:** 10.3390/cells12101421

**Published:** 2023-05-18

**Authors:** Michael G. Rizzo, Thomas M. Best, Johnny Huard, Marc Philippon, Francis Hornicek, Zhenfeng Duan, Anthony J. Griswold, Lee D. Kaplan, Joshua M. Hare, Dimitrios Kouroupis

**Affiliations:** 1 Department of Orthopedics, UHealth Sports Medicine Institute, University of Miami Miller School of Medicine, Miami, FL 33146, USA; teamrizzo@med.miami.edu (M.G.R.); txb440@med.miami.edu (T.M.B.); 2 Center for Regenerative and Personalized Medicine (CRPM), Steadman Philippon Research Institute, Vail, CO 81657, USA; m@mjphilippon.com (M.P.); 3 Department of Orthopedics, Sarcoma Biology Laboratory, Sylvester Comprehensive Cancer Center, University of Miami Miller School of Medicine, Miami, FL 33136, USA; fjh21@med.miami.edu (F.H.); zxd221@med.miami.edu (Z.D.); 4 John P. Hussman Institute for Human Genomics, University of Miami Miller School of Medicine, Miami, FL 33136, USA; agriswold@med.miami.edu; 5 Interdisciplinary Stem Cell Institute, University of Miami Miller School of Medicine, Miami, FL 33101, USA; 6 Diabetes Research Institute, Cell Transplant Center, University of Miami Miller School of Medicine, Miami, FL 33136, USA

**Keywords:** osteoarthritis, mesenchymal stem/stromal cells, senolytics, cellular senescence, extracellular vesicles, senescence-associated secretory phenotype, inflammation, cell-based therapies

## Abstract

Osteoarthritis (OA) is the most common cause of disability worldwide among the elderly. Alarmingly, the incidence of OA in individuals less than 40 years of age is rising, likely due to the increase in obesity and post-traumatic osteoarthritis (PTOA). In recent years, due to a better understanding of the underlying pathophysiology of OA, several potential therapeutic approaches targeting specific molecular pathways have been identified. In particular, the role of inflammation and the immune system has been increasingly recognized as important in a variety of musculoskeletal diseases, including OA. Similarly, higher levels of host cellular senescence, characterized by cessation of cell division and the secretion of a senescence-associated secretory phenotype (SASP) within the local tissue microenvironments, have also been linked to OA and its progression. New advances in the field, including stem cell therapies and senolytics, are emerging with the goal of slowing disease progression. Mesenchymal stem/stromal cells (MSCs) are a subset of multipotent adult stem cells that have demonstrated the potential to modulate unchecked inflammation, reverse fibrosis, attenuate pain, and potentially treat patients with OA. Numerous studies have demonstrated the potential of MSC extracellular vesicles (EVs) as cell-free treatments that comply with FDA regulations. EVs, including exosomes and microvesicles, are released by numerous cell types and are increasingly recognized as playing a critical role in cell–cell communication in age-related diseases, including OA. Treatment strategies for OA are being developed that target senescent cells and the paracrine and autocrine secretions of SASP. This article highlights the encouraging potential for MSC or MSC-derived products alone or in combination with senolytics to control patient symptoms and potentially mitigate the progression of OA. We will also explore the application of genomic principles to the study of OA and the potential for the discovery of OA phenotypes that can motivate more precise patient-driven treatments.

## 1. The Role of Inflammation and Cellular Senescence in OA

Inflammation is a basic tenant in the body’s acute response to injury as well as the subsequent healing and regenerative processes [1]. Signs and symptoms of inflammation have been recognized for millennia, and Celsus, a Roman scholar in the first century A.D., is credited with the first recognized description of the so-called “tetrad of inflammation” involving calor (heat), dolor (pain), tumor (swelling), and rubor (redness) [2]. Inflammation, fibrosis, and cellular senescence have been implicated in many musculoskeletal diseases, including osteoarthritis (OA), tendinopathies, metaplastic processes, and numerous others [3,4,5,6,7,8]. 

There are three phases of inflammation: pro-inflammatory, tissue repair and regeneration, and remodeling and maturation [9]. During the pro-inflammatory phase, the body’s innate immune response is activated, and inflammatory cells are recruited to the site of injury; in the tissue repair and regeneration phase, the inflammatory response subsides, and cells transition to a reparative state; in the final stage, the repaired tissue matures, and inflammatory cells either egress or undergo apoptosis to terminate the inflammatory response. Unchecked inflammation is associated with numerous consequences that can result clinically in chronic pain, edema, and loss of function [10].

Tissue repair begins with the influx of pro-inflammatory cells as part of the innate immune response [11,12]. The first cells to arrive at the site of inflammation are neutrophils, which phagocytose (ingest) and kill invading microorganisms through the release of enzymes and reactive oxygen species [13]. Neutrophils are short-lived cells and are typically only present at the site of inflammation for a few days before undergoing apoptosis (programmed cell death) [14]. Monocytes are additionally attracted to areas of active inflammation, where they differentiate into macrophages [11]. Macrophages have two main polarization states: the more classically described M1 macrophages are pro-inflammatory cells that produce inflammatory cytokines, including tumor necrosis factor-α (TNF-α) and interleukin (IL)-6, that phagocytose invading microorganisms, debris, and damaged tissue through the release of enzymes such as matrix metalloproteinases (MMPs) [15]. Alternatively, polarized macrophages (M2) play a more anti-inflammatory and regenerative role by producing transforming growth factor-β1 (TGF-β1) and fibronectin [11,16]. An imbalance between the M1 and M2 phenotypes can lead to a progressive cycle of persistent/chronic inflammation with increased expression of TGF-β1 and eventual fibrosis [17,18,19].

Chronic states of inflammation (e.g., metabolic syndrome), where synovitis is often present in the knees of patients with OA, have been increasingly recognized as an important contributor to disease pathogenesis. Patients with knee OA and metabolic syndrome have greater morbidity associated with their OA compared with patients with fewer comorbidities and no metabolic syndrome [20]. As early as 1953, it was recognized that the pathological process of OA was not simply degeneration but rather failed attempts at healing: “What is so damaging in osteoarthritis seems to be not the degeneration of the cartilage but the vigorous and persistent attempt at repair, an attempt which aggravates the already disordered function of the joint” [21]. Interestingly, the infrapatellar fat pad and synovium have increasingly been shown to play an orchestrated immunomodulatory role and together may contribute to the chronic state of inflammation typically seen in some patients with knee OA [22]. The degree of OA pain and disease severity have also been correlated with the extent of synovitis [23,24], and some studies even suggest the synovium may extend the articular cartilage (AC) degradation process [6,25,26]. 

Increasingly, the infrapatellar fat pad (IFP) has been implicated in the pathogenesis of OA. Heilmeier et al. observed that after an acute ACL injury, the IFP releases inflammatory cytokines that contribute to a sustained inflammatory response, often for several months [27]. Adipocytes within the IFP have been shown to secrete pro-inflammatory cytokines such as IL-1β and metalloproteases, in addition to hormones such as leptin and adiponectin, into the synovial fluid, which can contribute to inflammation within the joint, resulting in AC damage and degradation [28,29,30,31,32]. Leptin has additionally been shown to have a catabolic effect on AC and can activate M1 macrophages already present within the IFP through the upregulation of interleukins and nitric oxide [33,34].

**Figure 1:**
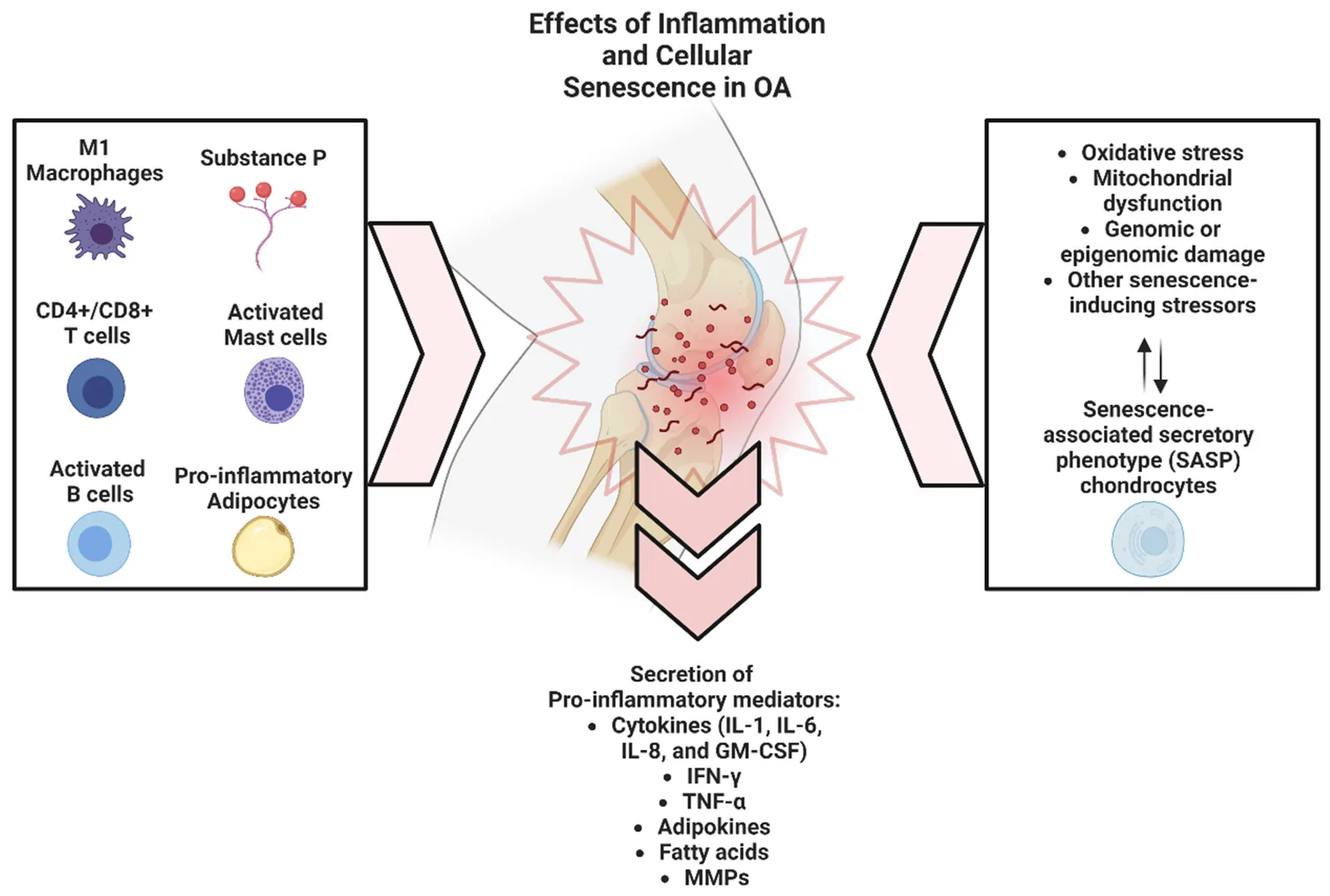
Effects of inflammation and cellular senescence in OA.

In addition to the resident M1 macrophages, various circulating immune cells, including CD4 and CD8 T cells, B cells, mast cells, and additional macrophages, can be recruited into the IFP and synovium through the action of pro-inflammatory mediators such as prostaglandins, IL-6, and IL-8 [35,36]. Substance P (SP), a neuropeptide produced by nociceptive nerve fibers, can also contribute to immune cell recruitment by inducing vasodilation of peripheral vessels, allowing for the extravasation of these immune cells [28]. Accordingly, the levels of these cells, such as CD4 T, have been correlated with pain scores [4]. Ultimately, the inflammatory state of a diseased joint represents a complex interplay between resident and circulating immune cells (Figure 1).

Cellular senescence has been implicated in multiple musculoskeletal diseases, including OA and osteoporosis [7]. Increased numbers of senescent osteocytes have been observed in both murine and human models [37,38], and induction of senescence in murine and human osteocytes has been shown to increase RANKL-dependent bone resorption [39]. We have recently shown that the elimination of senescent cells with senolytic treatment can improve bone health in a progeria animal model [40]. Senescent cells have also been shown to accumulate in osteoarthritic joints [41,42,43]. Studies have shown that senescent chondrocytes accumulate in OA joints, and their presence is positively correlated with the severity of the disease [41,44]. Senescent chondrocytes secrete SASP factors, which can cause an imbalance between cartilage synthesis and degradation, leading to structural dysfunction in the joint. The accumulation of senescent cells in OA joints has been linked to oxidative stress, mitochondrial dysfunction, genomic or epigenomic damage, or other senescence-inducing stressors that perpetuate the accumulation of additional senescent cells (Figure 1) [45]. In addition, senescent cells have been found in the subchondral bone of aged mice with surgically induced osteoarthritis (OA), while SASP has been implicated in age-related trabecular and cortical bone loss [37,46]. Senescent cell-associated extracellular vesicles serve as markers of OA and its response to therapy. The clearance of senescent cells has been shown to decrease the rate of post-traumatic OA in a murine model, suggesting that senescent cells may play a key role in the pathogenesis of post-traumatic OA [46]. It has been proposed that SASP products enhance the pro-inflammatory state of M1 macrophages, which can contribute to increased inflammation and cartilage degradation [7,47]. Accordingly, targeting senescent cells and their EV-derived cargo may represent a potential therapeutic approach for certain OA phenotypes.

## 2. Efficacy and Limitations of Standard-of-Care Modalities in Our Current Treatment Approaches for OA

The mainstay of conservative treatment typically involves oral or topical medications such as analgesics and anti-inflammatories, physical therapy, and injections. The primary role of analgesics, distinct from anti-inflammatories, is to relieve pain, but these often do not result in significant alteration of disease processes. Examples include lidocaine, which functions by blocking sodium and potassium ion channels on cell membranes [48]. Another example is acetaminophen, which is thought to function through the cyclooxygenase (COX) pathways primarily in the central nervous system and peripheral tissues, although its exact mechanism of action is still debated [49,50]. Anti-inflammatories can be categorized as steroidal or non-steroidal (NSAIDs). Both serve to decrease inflammation, although the pathways through which they accomplish this task vary. Steroids—specifically corticosteroids—bind to intracellular receptors to modulate gene expression and reduce the production of pro-inflammatory molecules [51,52]. NSAIDs, conversely, primarily inhibit COX, which reduces the synthesis of pro-inflammatory molecules such as prostaglandins [53].

Injectable agents, including corticosteroids, hyaluronic acid (HA), and platelet-rich plasma (PRP), have been shown to provide relief for symptomatic OA, but their efficacy varies [52]. Corticosteroids have been discussed above, but local administration of the drug provides targeted relief and may reduce the risk of systemic complications. HA is a naturally occurring glycosaminoglycan that is produced in joints to lubricate AC and increase the viscosity of synovial fluid. Injection of HA, also known as viscosupplementation, is intended to improve the lubrication of diseased joints [52]. PRP is prepared from a patient’s blood and contains a high concentration of activated platelets, which release growth factors that are believed to aid in healing and tissue regeneration [54]. The rationale for PRP use is strong, as it is easy to manufacture and administer, it does not show major adverse effects, and it is less aggressive than other therapeutic options (i.e., corticosteroids). Methodologies to prepare PRP vary, affecting clinical outcomes upon infusion in vivo. Specifically, PRP can be prepared by single centrifugation, double centrifugation, or blood selective filtration procedures, whereas platelets can be ex vivo activated mechanically with freeze–thawing cycles, chemically with thrombin or calcium chloride, or endogenously [55]. Important variables that affect therapeutic outcomes are (1) the ratio of platelets in PRP to platelets in whole blood, (2) the presence/absence of white blood cells, and (3) the method of PRP activation. On this basis, in order to optimize clinical efficacy for PRP, a standardized description of platelet-derived product characteristics has been proposed (reviewed in [56]).

Despite the availability of conservative measures, they have not been shown to have a sustained effect in the long-term mitigation of OA symptoms or disease progression and can have deleterious effects when prescribed over long durations [57]. NSAIDs, in particular, can lead to increases in blood pressure, cause gastric ulcers, and even lead to acute kidney failure, stroke, or myocardial infarction [58]. Chronic systemic steroid use can lead to osteoporosis, osteonecrosis, Cushing’s disease, adrenal insufficiency, hyperlipidemia, and many other effects [59]. Intra-articular injection of steroids has been shown to accelerate tissue damage and AC degeneration, so these are generally used judiciously [60,61,62]. Further, the use of intra-articular steroid injections has been shown to increase the risk of prosthetic joint infections in patients who do undergo total joint arthroplasty [63,64]. HA injections are relatively safe, with the main adverse reaction being a temporary local inflammatory reaction to the agent [52]. Similarly, PRP has been shown to only cause a focal increase in swelling and inflammation in the immediate period following injection.

Physical therapy (PT) has been used as a conservative therapy for most musculoskeletal pathologies and is often among the first conservative modalities utilized, alone or in combination with any of the above. PT has shown some effectiveness in reducing pain and improving function in hip and knee OA [65,66], yet recent evidence suggests that PT might not be superior to a sham intervention [67]. However, exercise and weight loss have been shown to have a positive impact on OA, so PT may assist in developing good exercise habits to support these efforts [66]. Articular cartilage heals best with a gradual increase in loading over time. Too little loading inhibits repair, but loading the joint too much too early can damage the healing cartilage. 

While there are a variety of conservative modalities available, these treatments do not significantly alter the course of the disease. Indeed, 52.2% of males and 50.6% of females with symptomatic knee osteoarthritis will eventually go on to receive a total knee arthroplasty [68]. Thus, there is a significant need for novel therapies that, ideally, provide disease-modifying activity. 

## 3. Investigation of Genetic Factors Contributing to OA

Given the noted complexity and heterogeneity of OA, elucidating the underlying genetic factors contributing to the disease has been challenging. Evidence for the role of genetics in OA has been previously noted by epidemiologic and family studies [69,70], with heritability estimates ranging from 39% to 79% [71,72]. Candidate gene studies have been conducted on structural molecules, including those involved in extracellular matrix (ECM) genes that affect the inflammatory cascade, such as cytokines, genes that influence joint development, proteins that impact mitochondrial apoptosis, and many others [69,72]. As the costs of genomic analyses have decreased and large community-based cohorts have been collected, there has been a rapid increase in the number of studies set up to identify genetic markers that could provide important insights for disease screening and potential treatment targets [73]. In particular, the largest genome-wide association study (GWAS) in more than 77,000 cases and 375,000 controls in the UK Biobank identified genes that are targets of existing therapeutics, including *TGFB1* (transforming growth factor beta 1), *FGF18* (fibroblast growth factor 18), *CTSK* (cathepsin K), and *IL11* (interleukin 11) [74]. Overall, across a number of such GWAS studies, more than 120 genetic variants in 95 loci have been associated with OA [69]. These molecular studies have in turn spurred investigations of genetic animal models of OA, with more than 20 mutant mouse strains identified that recapitulate some OA features [75].

Critically, while these genetic studies have enhanced knowledge related to the genes and molecular pathways contributing to OA, there has been a concurrent effort to expand understanding of the functional roles of these genes and molecules in OA pathogenesis. These include transcriptomic analyses in human tissues relevant to OA, including cartilage and subchondral bone [76] and cell-free RNA from synovial fluid [77]. Moreover, quantitative trait analyses have linked several OA-associated genetic variants with gene expression alterations, further enhancing our understanding of the mechanisms by which risk is conferred [78]. As technologies continue to evolve, our understanding of the cell-specific effects contributing to the heterogeneity of OA has improved. For example, single-cell transcriptomic approaches provide unprecedented resolution and insights into the heterogeneity of cellular activities in OA, including in the cartilage [79] and the fat pad of animal models [80]. Continued investigation of the genetic and functional mechanisms of OA in human tissue and in animal models of OA will further establish links between genetics, transcriptomics, and cellular functions to refine the pathophysiology of disease and suggest potential novel therapeutic applications.

## 4. Mesenchymal Stem Cells (MSCs) and Mesenchymal Stem Cell-Derived Extracellular Vesicles as Therapeutic Modalities

### 4.1. MSCs Therapeutic Capacity in Inflammation and Pain 

MSCs are multipotent adult stem cells that can be isolated from many tissues and have been shown to have local anti-inflammatory and analgesic effects [81]. They were first identified by Friedenstein in 1974 as multipotent cells capable of conferring a microenvironment in tissues [82]. Later research suggested that they also have a regenerative capacity, particularly in vascular maintenance and repair [83]. This role was expanded to include immunomodulatory effects in subsequent studies (reviewed in [84]). Initially, it was believed that MSCs exerted their activity through cell-to-cell interactions with immune system cell populations [85]. In vitro studies have suggested that MSCs can upregulate the expression of intercellular adhesion molecule-1 (ICAM-1) and vascular cell adhesion molecule-1 (VCAM-1), which are important for immune-cell trafficking and adhesion to tissues [86]. Through upregulation of these proteins, MSCs instead adhere to tissues and prevent immune cells from adhering and causing inflammation. Over the last decade, much work has been devoted to profiling the regenerative qualities of MSCs, indicating paracrine activity is their primary mode of action [87]. On this basis, MSCs have been shown to affect tissues through paracrine activity by secreting cytokines, exosomes, and microRNAs [88]. Deemed “injury drugstores”, MSCs have been shown to have anti-inflammatory, anti-fibrotic, and analgesic therapeutic activity, all of which could impart benefit to patients with OA [88]. 

The anti-inflammatory action of MSCs results from interactions with both the innate and adaptive immune systems. The innate immune system contains several different cell lines, with the monocyte/macrophage lineage being a central player [89]. MSCs have been shown to secrete various proteins, including prostaglandin E2 (PGE2), IL-6, and granulocyte-macrophage colony-stimulating factor (GM-CSF), which inhibit the polarization of monocytes into M1 macrophages and permit M2 macrophages to dominate [90,91,92]. Mainly, MSCs polarize activated pro-inflammatory (M1) macrophages into an M2 anti-inflammatory phenotype through a PGE2-dependent mechanism [93,94,95,96]. MSCs have also been shown to inhibit neutrophil apoptosis and their pro-inflammatory respiratory burst through IL-6-mediated pathways [97]. MSCs can also inhibit mast cell degranulation and production of TNF-α by producing PGE2 and upregulating COX-2 [98]. MSCs primarily affect the adaptive immune system through interactions with B and T lymphocytes. By binding directly to PD-1 receptors on B cells and secreting anti-inflammatory factors such as TGF-β and galectin 9, MSCs prevent the activation of B cells into plasma cells [90,99]. MSCs also inhibit both CD4+ (helper) and CD8+ (cytotoxic) T cells through cell-cell contact and paracrine mechanisms, including direct binding to programmed cell death protein 1 (PD-1), and the secretion of multiple factors, such as PGE2, TGF-β, and galectin-1 [90,99,100]. MSCs can induce the differentiation of CD4+ T cells into CD4^+^CD25^(high)^FoxP3^+^ T Regs [101,102,103]. Specifically, MSCs augment T Regs-mediated immunosuppression through HLA-G5, B7-H4, IL-10 secretion, and up-regulation of PD-1 receptors on T Regs [104,105]. Unregulated inflammation is a primary driver of fibrosis, and MSCs exert their effects through the attenuation of these inflammatory pathways. Specifically, this downregulation of inflammation occurs through decreased expression of pro-inflammatory molecules such as TNF-α and IL-1β, shifting macrophage polarization toward the M2 anti-inflammatory phenotype, and inhibiting B cell infiltration [106]. Thus, MSCs exhibit strong anti-inflammatory, anti-fibrotic, and analgesic effects through direct interaction with the effector cells of the innate and adaptive immune systems. 

It is widely accepted that MSC populations within different niches and tissues are highly heterogeneous. However, previous studies suggest that distinct MSC subpopulations possess superior functionality, especially for immunomodulation (reviewed in [107]). Investigations have helped distinguish subpopulations within crude MSCs based on the expression of the pericyte-related marker CD146. In general, MSCs share similar characteristics with pericytes that are found in the perivascular niche, including the expression of CD146, NG2, and PDGF-Rβ, as well as the ability to differentiate into multiple cell types and play a role in maintaining tissue homeostasis and modulating the immune system [108,109,110,111,112]. There is evidence that MSCs and pericytes may have a shared developmental origin and that MSCs may differentiate into pericytes under certain conditions [83,113]. Based on this, Bowles et al. determined that the CD146+ MSC subpopulation is correlated with an innately higher immunomodulatory and secretory capacity [114]. In a rat model of inflammation and fibrosis, the study showed that treatment with CD146+ MSCs resulted in greater therapeutic efficacy compared to the CD146− subpopulation, promoting M2 macrophage polarization and reducing inflammation and fibrosis in the synovium and fat pad tissues [114]. These findings suggest that the CD146+ subpopulation is the main mediator of MSC therapeutic effects observed with crude MSC preparations and may have potential as a therapeutic option for OA patients whose phenotype is characterized by joint and periarticular inflammation and fibrosis.

CD10, also known as neprilysin, is a surface enzyme expressed in MSCs and is involved in the degradation of various signaling molecules, including the molecule of pain substance P (SP), a compound secreted by sensory nerve fibers in the synovium and interstitial fluid and associated with nociceptive pathways of pain [115]. In a recent study, Kouroupis et al. investigated the role of CD10 in MSCs and its effects on SP degradation [116]. CD10 was highly enriched in IFP-MSCs after exposure to pro-inflammatory and pro-fibrotic conditions, leading to a significant reduction in SP levels. Inhibition of CD10 enzymatic activity with thiorphan abrogated this effect, supporting a CD10-dependent mechanism of SP degradation. Furthermore, a general correlation between CD10 expression and SP levels was observed in both naive and stimulated IFP-MSCs and bone marrow-derived MSCs (BM-MSCs). Supernatants obtained from stimulated MSC cultures also contained CD10 and exhibited SP degrading activity, suggesting that CD10 may be released by MSCs in the form of extracellular vesicles. In a subsequent study, the same group found that preparing IFP-MSCs in a regulatory-compliant medium resulted in higher CD10 and CD146 expression as well as improved degradation of SP in vitro and in vivo, with reversal of synovitis and fibrosis even at lower cell doses [117]. These findings suggest that CD10 expression and activity in MSCs may contribute to their anti-inflammatory, analgesic, and anti-fibrotic effects, potentially through the degradation of SP. Elevated synovial levels of SP have previously been linked to the severity of pain in knee OA and the magnitude of pain relief following arthroplasty; accordingly, targeting SP with MSCs may provide a novel treatment for knee OA [118].

### 4.2. Efficacy and Limitations of These Alternative Therapeutic Modalities in Preclinical and Proof-of-Concept Clinical Trials Aimed at Modulating OA

There is a growing body of research examining the use of MSCs for the treatment of OA, both in preclinical and clinical studies. In preclinical studies, MSCs have been evaluated as a potential treatment for cartilage damage, where they may differentiate into functional chondrocytes and contribute to the repair and regeneration of the damaged tissue (Table 1) [119,120,121]. While this approach has shown some promise, there are limitations to the use of MSCs for cartilage regeneration, including the fact that the injected cells do not directly participate in the repair process and the regenerated tissue may not fully resemble native AC and may have different structural characteristics [120,122]. There is a growing body of research examining the use of MSCs for the treatment of OA, both in preclinical and clinical studies. In preclinical studies, MSCs have been evaluated as a potential treatment for cartilage damage, where they may differentiate into functional cartilage cells and contribute to the repair and regeneration of the damaged tissue [119,120,121,123,124,125]. While this approach has shown some promise, there are limitations to the use of MSCs for cartilage regeneration, including the fact that the resulting tissue may not fully resemble native AC and may have different structural characteristics [120,122]. Further preclinical studies using MSCs for AC regeneration are described in [126].

Despite these limitations, clinical studies have shown that MSC treatment may offer promise to patients with OA. For example, Pak has described a case series where implantation of autologous adipose-derived MSCs into hips with osteonecrosis and knees with OA showed bone and cartilage regeneration as measured on MRI [137]. Other studies performed by Koh et al. and Freitag et al. have demonstrated the efficacy of using autologous adipose-derived MSCs in combination with arthroscopic microfracture or abrasion for the treatment of knee cartilage defects and generalized OA with some success [138,139,140]. Allogeneic MSCs have also been studied for the treatment of knee cartilage defects. In a phase I clinical trial, de Windt et al. demonstrated the utility of allogeneic MSCs mixed with autologous cartilage-derived cells in improving clinical outcomes and the appearance of cartilage lesions on MRI and second-look arthroscopy, suggesting AC regeneration [141]. In a multicenter, double-blind, randomized controlled trial, Vangsness et al. found that allogeneic MSC injections following partial medial meniscectomy resulted in significantly increased meniscal volume and reduced pain in patients with knee OA compared to treatment with hyaluronic acid [142]. These outcomes suggest that allogeneic MSCs may assist in the meniscal regeneration process and additionally provide analgesia and possibly AC restoration [142]. Similarly, Vega et al. showed the effectiveness of allogenic MSC injections in improving patient-reported outcomes (PROs) and cartilage appearance on MRI compared to hyaluronic acid [143]. Further clinical studies using MSCs for AC regeneration have also been reported [126,144]. Despite promising initial results, a significant amount of heterogeneity exists regarding the dosing of MSCs. Several studies have evaluated various doses, but a consistent dosing protocol or dose-dependent effect has yet to be confirmed [126,134,145,146,147,148]. Interestingly, in a review on pre-clinical MSC dosing, Wang et al. found a positive correlation between MSC dose and animal weight but were unable to conclude an optimal dosing regimen [126]. Furthermore, while MSCs have generally demonstrated anabolic effects on AC, some studies have reported no significant improvement in AC reconstruction and only improvements in pain scores, suggesting further research is needed to fully understand MSCs’ potential benefits [128]. 

Overall, MSCs show promise as a treatment for various musculoskeletal conditions, but additional long-term studies are needed to assess their continued efficacy. Concerns about potential side effects of cell-based therapies have slowed their adoption into wider clinical use, despite no adverse events being reported in any of the aforementioned studies. Indeed, all were performed outside the United States, as cell-based therapy is not yet approved by the U.S. Food and Drug Administration (FDA) due to concerns about malignant transformation of the cells or hypersensitivity reactions. To determine whether MSCs were associated with adverse events in a larger population, Centeno et al. reviewed the records of 2372 patients who received MSC injections at 18 facilities in the United States or Australia [149]. Interestingly, they found that only seven patients developed a new malignancy, which was a lower rate than the general untreated population. Adverse events were reported in 325 patients (13.7%), and the most frequent were pain post-procedure (3.9%) and pain due to accelerating degenerative joint disease (3.8%). These results suggest that the use of MSCs is safe, but further studies in more diverse populations and longer-term follow-up are needed. 

### 4.3. Application of MSC-Derived Extracellular Vesicles

The concern regarding the potential side effects and increased immunogenicity of cell-based therapies has led researchers to pursue cell-free treatments focusing on the MSC secretome, especially their extracellular vesicles (EVs) [150]. While MSCs do secrete cytokines and chemokines directly, they also exhibit their paracrine effects via EVs, especially exosomes. Exosomes are nanosized (50–200 nm) EVs generated via the endosomal pathway [151], and secreted by MSCs in response to their surrounding milieu. They serve as vehicles for cellular export products, including lipids, proteins, and RNAs (mRNAs and miRNAs), and can modulate the function of other cells at proximal or distal sites [152]. Because EV composition is believed to reflect the characteristics of their parent cells, EVs should convey many of the benefits of MSCs if prepared properly [152]. To date, studies have isolated and characterized exosomes from various MSC sources (i.e., bone marrow, umbilical cord, and adipose tissues), confirming their strong anti-inflammatory, anti-fibrotic, and angiogenesis-remodeling capacities [152]. 

Many studies have found that MSC-EVs have strong immunomodulatory properties, particularly through the action of miRNAs, which may be able to target the immune system and modulate angiogenesis [153,154,155]. Our laboratory has demonstrated that MSC-EVs isolated from a CD146+ subpopulation possess enhanced anti-inflammatory capabilities through the high expression of immunomodulatory miRNAs [154]. Another study found that IFP-MSCs had an immunomodulatory secretome with strong miRNA attributes that were able to reduce synoviocyte and macrophage proliferation and inflammation-related molecular profiles, as well as reduce the secretion of pro-inflammatory molecules when stimulated in vitro [155]. In an acute synovial/IFP inflammation rat model, MSC-EV’s therapeutic treatment resulted in robust macrophage polarization towards an anti-inflammatory therapeutic M2 phenotype within the synovium/IFP tissues [155]. These findings suggest that MSC-EVs may have therapeutic potential for the treatment and identification of inflammation and fibrosis. Additionally, assessment of levels of EVs may help in defining the disease state in neoplastic and non-neoplastic conditions [156].

In addition to their immunomodulatory effects, MSC-EVs have been shown to play a role in regulating cell proliferation, particularly in the context of AC repair and regeneration [157]. In vitro studies have demonstrated the ability of MSC-EVs to promote chondrocyte proliferation and inhibit chondrocyte apoptosis [104,130,135,158,159,160]. One of the main mechanisms by which MSC-EVs promote chondrocyte proliferation is through the transfer of miRNAs, which regulate gene expression and modulate various signaling pathways involved in cell proliferation. For example, MSC-EVs have been shown to transfer miRNAs, which can promote chondrocyte proliferation through the regulation of CDH11, NF-kB, ROCK1, TLR9, and Wnt5a [130,135,158,159,160,161]. Additionally, miRNAs from MSC-EVs have also been shown to inhibit chondrocyte apoptosis in OA by inhibiting the expression of pro-apoptotic proteins such as HMGB1, IL-1β, and RUNX2 [162,163,164]. Furthermore, MSC-EVs have been shown to stimulate the production of ECM components in chondrocytes, including proteoglycans and collagen. For example, exosomes derived from MSCs have been shown to increase the production of aggrecan and collagen II in chondrocytes [165]. MSC-EVs may also modulate the activity of enzymes involved in ECM synthesis and degradation, such as aggrecanases and MMPs, to promote ECM production and inhibit degradation in chondrocytes [157,165,166]. These effects of MSC-EVs on ECM production may contribute to their potential therapeutic effects in OA.

Animal models have also been employed to further characterize the beneficial effects of MSC-EVs and establish their safety profile. Qi et al. found that the implantation of MSC-EVs into large bone defects in a rat model of osteoporosis enhanced bone regeneration and angiogenesis in a dose-dependent manner [167]. Zhang et al. also observed beneficial effects in rats with surgically-induced osteochondral defects of the knee [129]. One extremity was treated with intra-articular MSC-EVs and the other with a buffered control immediately following surgery and then weekly for 12 weeks following surgery. They found that there was a complete restoration of cartilage and bone with healthy hyaline tissue closely resembling age-matched controls, whereas only fibrous tissue was found in the buffered-solution control group. Khatab et al. found similar results in a mouse model of collagenase-induced OA [133]. Intra-articular injections of MSC-EVs or MSCs improved pain compared to the control group and did not cause AC damage. Interestingly, there was no difference between the effects of MSC-EVs and MSCs, suggesting that MSC-EVs may provide a convenient and effective alternative to traditional MSCs for the treatment of OA without the same regulatory hurdles as whole-cell treatments.

Collectively, results to date show promise and suggest that the immunomodulatory effects of MSC-EVs may induce AC regeneration in vivo, but more studies are needed to expand these applications clinically in humans. MSC-EVs are attractive candidates for treatment because they are easier to regulate, are more stable, and pose fewer safety risks than cell-based treatments, such as microvascular occlusion, compared with other cell-based treatments [168,169].

## 5. Cellular Senescence and Senolytic Agents

### 5.1. Mechanisms and Stimuli for Cellular Senescence in Chronic Inflammation

Cellular senescence was first observed by Hayflick et al. in 1961 and was initially thought to be an in vitro phenomenon where cultured human fibroblasts lost their ability to replicate [170,171]. However, recent research has demonstrated that cellular senescence includes a diverse set of cellular states that are caused by significant cellular stress such as DNA damage or telomere erosion [7,172]. This leads to the arrest of the cell cycle and the ability to impact local environments through the senescence-associated secretory phenotype (SASP) [7,172]. It is believed that cellular senescence evolved as a mechanism to prevent unregulated cell replication, such as cancer, but similar to unregulated inflammation, persistent or overwhelming senescence can have significant adverse effects on the musculoskeletal system. SASP can likewise have both positive and detrimental effects on the organism, depending on the host environment [173]. It can combat cancer by inducing a pro-inflammatory environment and even triggering senescence in neighboring cells [174,175,176,177], and yet concurrently, the SASP can promote tumorigenesis and lead to chronic inflammation [178,179,180]. Therefore, cellular senescence is a delicate balance, with slight alterations having the potential to cause widespread changes and states of disease. 

Cellular senescence can be triggered through two major signaling pathways: the p53/p21 pathway and the p16INK4a/Rb pathway [163]. Previous studies have observed higher expression of p16 in older chondrocytes in both mice and humans, and selective elimination of p16 has been shown to improve the life span of mice [181,182]. The p53/p21 pathway is activated in response to DNA damage or other stresses and leads to the activation of the p21 protein, which inhibits cell cycle progression [183]. The p16INK4a/Rb pathway is activated by DNA damage, oxidative stress, telomere shortening, inflammation, and aging [184,185]. Activation of p16INK4a inhibits cell cycle progression and promotes senescence by preventing the inactivation of Rb by CDK4 or CDK6 [186]. While both pathways are activated by stress, the p16/pRB pathway has largely been found to be more irreversible [186]. Senescent cells activate various anti-apoptotic pathways (SCAPs) such as B cell lymphoma family inhibitors, PI3K/Akt pathways, p53/p21Cip1/serpine pathways, HIF-1α, and HSP-90 to protect themselves from proapoptotic SASP molecules [179,187,188]. Furthermore, studies have previously shown that a deficiency in autophagy can lead to senescence in articular chondrocytes [189,190,191]. The mTOR signaling pathway is activated downstream of PI3 kinase and Akt kinase to inhibit autophagy. Overexpression of mTOR has been observed in chondrocytes of OA patients and mouse models [192,193], and the PI3K/Akt/mTOR pathway has been shown to regulate chondrocyte death in a rat model, thus implicating this pathway in the pathogenesis of OA [194,195]. Increased autophagy has been shown to postpone cellular senescence by inhibiting the PI3K/Akt/mTOR signaling pathway. Beclin-1 is a key protein in the pathway, and it promotes the formation of autophagosomes to induce autophagy by inhibiting the PI3K/Akt/mTOR pathway [196]. Pitx1 has also been associated with cartilage degeneration, and a recent study found that overexpression of Pitx1 inhibits chondrocyte senescence by promoting autophagy by increasing SIRT1 and Beclin-1, thus inhibiting the PI3K/Akt/mTOR pathway [197,198]. Overall, there is a complex network of signaling pathways involved in cellular senescence and OA progression. Understanding these pathways and identifying potential targets for intervention may assist in mitigating the progression of OA. 

On this basis, cellular senescence is a process closely tied to aging and chronic inflammation. It arises from cellular stress such as oxidative stress, DNA damage, and oncogene expression [199,200,201]. Oxidative stress causes the accumulation of reactive oxygen species (ROS) within cells, which can damage cellular components such as DNA and proteins, so cellular senescence may have evolved to curtail the propagation of damaged cells. Indeed, researchers have observed that an increase in intracellular ROS can stimulate senescence in fibroblasts [202,203]. Similarly, direct DNA damage can also lead to senescence, but it can also cause apoptosis depending on a myriad of factors [204]. While apoptotic cells can be induced by similar stimuli, they will die and no longer be a factor; in contrast, senescent cells persist with an altered phenotype, which can contribute to the development and progression of musculoskeletal diseases. Specifically, the SASP has been implicated in perpetuating inflammation through the release of pro-inflammatory cytokines such as IL-1, IL-6, IL-8, and GM-CSF, among others [199]. These cytokines upregulate inflammation in surrounding tissues and also induce further senescence in neighboring cells. As a result, cellular senescence and chronic inflammation form a continuous cycle that leads to progressive degeneration and tissue destruction.

### 5.2. Senolytic Agents and Their Functionality in Cellular Senescence Clearance, Anti-Fibrotic, and Anti-Inflammatory Therapeutic Modalities

As the harmful effects of cellular senescence become better understood, there is an emerging need for therapeutics to either reduce SASP or remove senescent cells [205,206]. In a landmark proof-of-concept paper, Baker et al. demonstrated in 2011 that selectively eliminating senescent cells via a drug-inducible “suicide gene” improved age-related conditions in mice, including exercise intolerance, lordokyphosis, and cataracts, both in the early and late stages of life [207]. Later in 2015, Zhu et al. identified several drugs with senolytic activity, including the SRC/tyrosine kinase inhibitor dasatinib and the flavonoid quercetin [187]. While each of these drugs had senolytic activity on its own, the combination (commonly referred to as D + Q) had the greatest effect, showing increased longevity in mice and delayed onset of age-related conditions such as osteoporosis and loss of intervertebral disk proteoglycans, without any apparent adverse effects. Zhu et al. later identified additional senolytic agents, including the flavonoid Fisetin (F) and the BCL-2/BCL-W/BCL-X_L_ inhibitor, navitoclax (N) [208,209]. The discovery of these various agents has led to a series of studies examining their effects alone or in combination.

The harmful effects of senescent cells are believed to occur only when the level of senescence in an organism reaches a certain point, and they are time-dependent post-injury. In fact, the involvement of senescent cells early (acute) after the injury might be helpful for the repair process, while the involvement of senescent cells in chronic inflammation can be deleterious to the healing process. This so-called “Threshold Theory” of cellular senescence was observed by Xu et al., who found that the number of transplanted senescent cells needed to induce an age-like phenotype in mice was smaller in older mice than in younger ones [210]. They postulated that the reason for this effect is that while senescence in isolation or small amounts is an evolutionary response to limit tumorigenesis, in larger quantities, senescent cells can induce senescence in healthy cells even at a distance. Once senescence reaches this critical threshold, it enters a positive feedback loop, increasing the number of senescent cells and the harmful effects of the SASP. Moreover, Xu et al. demonstrated that this aging phenotype could be reversed through the intermittent administration of senolytics (D + Q), resulting in increased survival and reduced mortality hazard, suggesting that the level of senescence could be reduced to sub-threshold levels to alleviate its harmful effects [210].

Senolytics are under active investigation in a variety of fields and have been shown to have beneficial effects on diseases such as Alzheimer’s, Parkinson’s, cardiac disease, vascular disease, chronic kidney disease, diabetes, cancer, and COVID-19, among others [211,212]. In the musculoskeletal sector, there have been recent advances that highlight the potential of senolytics in the treatment of various musculoskeletal pathologies, including OA (Table 1). In a mouse model of age-related osteoporosis, the administration of D + Q led to a lower number of senescent cells, lower osteoclast activity, and higher femur cortical thickness [131]. In a mouse model of age-related muscle loss, mice that received D + Q had larger muscles and muscle fibers after 14 days of mechanical overload, suggesting that senolytics can improve muscle growth in older mice [213]. Fisetin has also been shown to have a beneficial effect on various musculoskeletal diseases, including osteoporosis, OA, and muscular dystrophies [214]. In a mouse model of osteoporosis, Fisetin improved bone mineral density [127,136], and in a mouse model of OA, it reduced cartilage degeneration, decreased subchondral bone plate thickness, and improved synovitis without causing any adverse effects [132]. In a mouse model of dystrophic muscle, treatment with Fisetin reduced the number of senescent macrophages and increased the number of healthy muscle cells, improving muscle phenotypes [215]. These results suggest that senescent macrophages may play a significant role in the development of muscle dystrophy by impacting the function of muscle stem cells and that the senolytic elimination of these cells may be a promising therapeutic strategy. We have recently shown that the elimination of senescent cells with senolytic treatment (Fisetin) can improve bone health in a progeria animal model that develops premature osteoporosis [40]. Together, these results suggest that senolytics may have the potential for use in the treatment of human age-related musculoskeletal disorders, including OA.

While senolytics have shown promise in the treatment of musculoskeletal diseases in animals, there have been limited applications in human trials. A phase 2 clinical trial of the senolytic UBX0101 found no significant improvement in pain or function for knee OA patients compared to the control group [216]. However, several studies have reported that UBX0101 only has weakly senolytic effects, so its use as the sole agent in this clinical trial may not have been sufficiently senolytic to produce an observable effect [42,212,217,218]. Additionally, multiple foods have been shown to have anti-inflammatory, anti-aging, and senolytic properties, such as resveratrol found in grapes and red wine and the spice turmeric [219,220]. Research into the effects of senolytics is still in its early stages, and more studies are needed to fully evaluate their potential and safety profile. Several clinical trials are currently underway examining the effects of senolytics on aging, cartilage degeneration, and OA [221,222,223,224]. The results of these studies will provide valuable insight into this expanding field.

## 6. MSCs and Senolytics: Combinatory Therapeutic Paradigms

Despite advances within the fields of MSCs and senolytics, there has been little research examining their combined effects. One of the challenges to the use of expanded MSCs is the presence of cellular senescence. The senescence of MSCs has been observed to limit their anti-inflammatory effects and even contribute to a pro-inflammatory state [225]. Malaise et al. showed that senescent MSCs were able to induce AC breakdown in mice, suggesting that senescent MSCs may play a role in the development of degenerative joint disease [226]. This raises the question of the effectiveness of MSC-based therapies when the MSC donor is older, as older patients with an increased number of senescent MSCs could experience negative effects from using that population of cells. Wang et al. observed this phenomenon in a murine model, where the implantation of MSCs from old donors caused physical dysfunction but not from young donors [227]. They further identified a subset of cells in the old donor group that had an abundance of senescent cells, suggesting that the observed dysfunction was due to the presence of senescent MSCs. Senescent MSCs, therefore, have reduced therapeutic potential and may even be harmful if transplanted into patients. We have also observed that during the expansion of MSCs isolated from adipose tissue and bone marrow, accumulation of senescent cells occurs [228].

**Figure 2:**
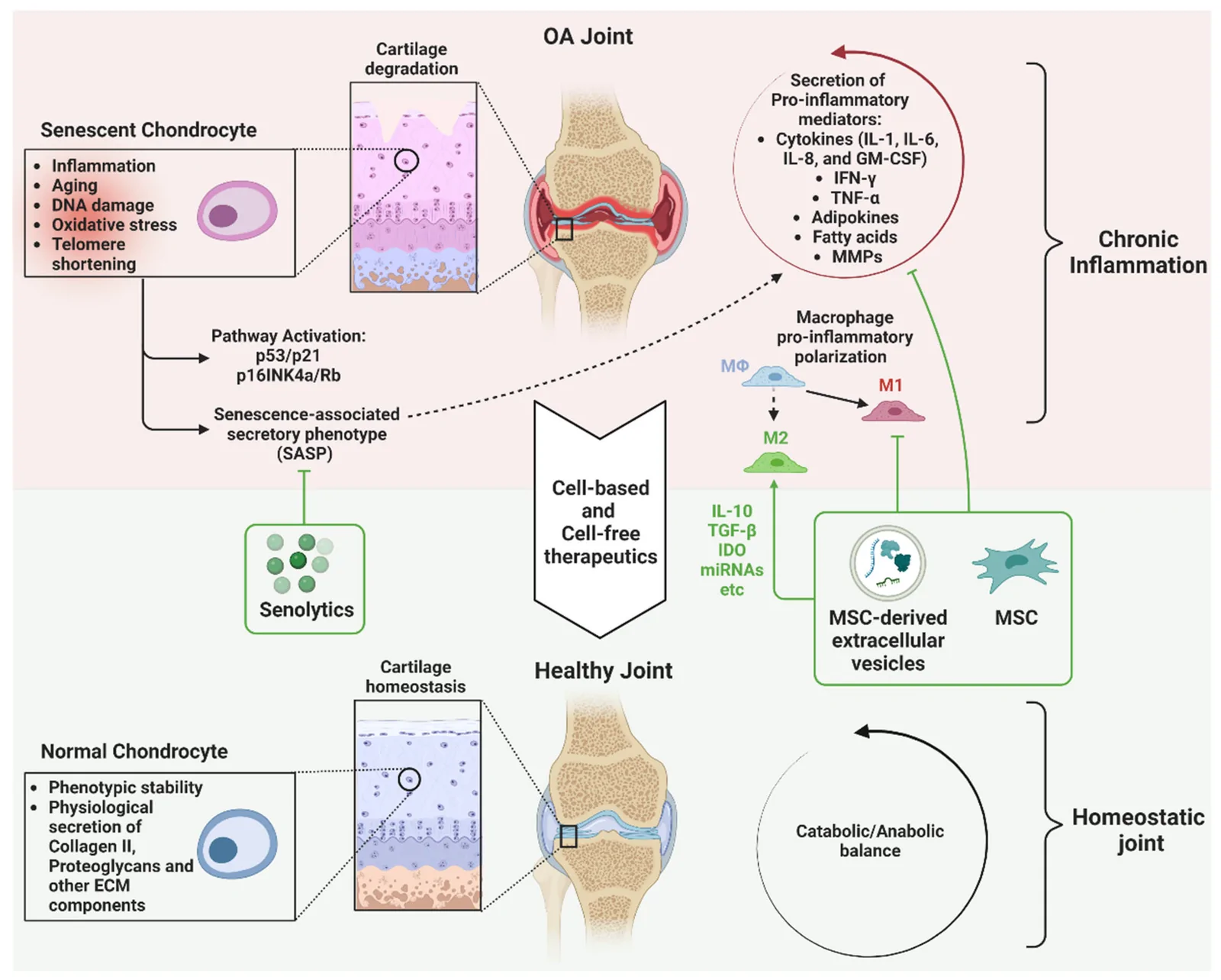
Effects of MSCs, MSC-derived EVs, and senolytic agents in the OA joint.

The use of senolytics with MSCs has the potential to improve the host’s condition and the efficacy of MSCs. There are several possibilities for the use of senolytics with MSCs (Figure 2). One possible application is to use senolytics in a host before MSC harvest to decrease the number of senescent MSCs and improve the yield and potency of the harvested cells. To our knowledge, no studies have yet been performed that analyze the yield and potency of MSCs harvested from a senolytic-treated host.

Another possibility is to treat harvested MSCs with senolytics during expansion and prior to reimplantation. Zhou et al. showed that treating MSCs from old mice with D + Q improved their osteogenic capacity [229]. They incubated the MSCs derived from younger or older mice with D + Q for 1 day before re-implantation and found that osteogenic activity was improved; moreover, D + Q only improved the osteogenic capacity for the MSCs from old mice and not those from young mice, suggesting that this improved function was due to the clearance of senescent cells.

A final option would be to use senolytics and MSCs in combination in a host to synergistically amplify their anti-inflammatory and regenerative effects. Both senolytics and MSCs have been shown to have therapeutic effects for musculoskeletal conditions, so their combination is likely to be effective as well. Senolytics could be used to clear senescent cells from the host and remove any potentially harmful senescent MSCs, while MSCs could be used to repair damaged tissues. This combination may be particularly useful in treating musculoskeletal conditions related to aging with chronic inflammation, as it could both improve the host’s condition and enhance the therapeutic potential of MSCs. The timing, volumes, and modes of administration will be critical to understanding the utility of this combination. 

## 7. Conclusions

MSCs, MSC-derived EVs, and senolytic agents show exciting potential for curtailing inflammation and may provide a novel treatment approach for OA (Figure 2). While significant work is underway examining each of these agents, there is a paucity of research examining them in combination. Preliminary studies suggest that senolytics can increase the yield and therapeutic activity of MSCs, but more research is needed to fully understand the potential of using these two modalities together. Further work is needed to deepen the understanding of the beneficial effects of MSCs, MSC-derived EVs, and senolytics and their synergies to establish appropriate dosing and potential risks. Ultimately, the use of MSCs and senolytics is a promising future direction of regenerative medicine to help slow down the progression of aging-related diseases, such as OA.

## Figures and Tables

**Table 1:** Preclinical studies using MSCs and/or senolytics in OA and bone loss animal models.

Reference	Animal Model	Therapeutic Group	Control Group	Follow-Up	Therapeutic Effect	Outcomes
[124]	White New Zealand rabbits as models of OA	Intraarticular injection of infrapatellar fat pad-derived MSCs	1 mL of medium without cells	16 and 20 weeks post-surgery	Benefit	Reduced cartilage degeneration, osteophyte formation, and subchondral sclerosis; improved cartilage quality.
[125]	Hartley-strain guinea pigs with spontaneous OA	Intra-articular injection of commercially available MSCs suspended in PBS or HA	PBS or HA alone	1, 3, and 5 weeks	Benefit	Partial cartilage repair, MSC migration, differentiation, and proliferation.
[127]	- Age-related bone loss in the mouse model - Ovariectomized bone loss mouse model - LPS-induced bone loss mouse model	Daily oral gavage with fisetin (5 to 50 mg/kg)	Vehicle	4 weeks	Benefit	- Fisetin treatment significantly prevented bone loss in estrogen deficiency and inflammation models of osteoporosis in mice. - Bone mineral density, micro-architecture parameters, and bone markers were positively modulated by fisetin.
[128]	Mono-iodoacetate-induced OA rat model	- Intra-articular injection of rat bone marrow-derived MSCs (1 × 10^6^) - Intra-articular injection of rat bone marrow mononuclear cells (10 × 10^6^)	Saline	4 weeks	Limited benefit	MSCs reduced pain, but there were no significant effects on cartilage damage, subchondral bone alterations, or synovial inflammation.
[129]	Surgically-induced OA rat model	Intra-articular injection of exosomes from human embryonic cells-derived MSCs (100 μg)	PBS	6 and 12 weeks	Benefit	Exosome treatment enhanced gross appearance, improved histological scores, and resulted in complete restoration of cartilage and subchondral bone with features resembling those of an age-matched unoperated control.
[123]	Mice with a human age-related osteoporosis model	Systemic injection of minimally expanded exogenous MSCs	Age-matched wild-type mice	24 weeks post-engraftment	Benefit	Increased bone formation, improved bone quality, and microarchitectural competence.
[130]	Surgically-induced OA rat model	- Intra-articular injection of exosomes from human synovial mesenchymal stem cells (SMSC-Exos, 10^11^ exosome particles/mL) - Intra-articular injection of exosomes from miR-140-5p-overexpressing human synovial mesenchymal stem cells (SMSC-140-Exos, 10^11^ exosome particles/mL)	Saline	12 weeks	Benefit	SMSC-140-Exos enhanced the proliferation and migration of articular chondrocytes without harming extracellular matrix secretion and prevented OA in a rat model.
[131]	Age-related bone loss mouse model	- Once-monthly treatments by oral gavage with dasatinib and quercetin (5 mg/kg and 50 mg/kg, respectively) - Daily administration with chow JAK 1/2 inhibitor, ruxolitinib (JAKi) (60 mg/kg)	Vehicle	2–4 months	Benefit	- Treatment with senolytics or the JAKi resulted in higher bone mass and strength and better bone microarchitecture than in vehicle-treated mice. - The beneficial effects of targeting senescent cells were due to lower bone resorption with either maintained (trabecular) or higher (cortical) bone formation as compared to vehicle-treated mice.
[132]	Surgically-induced OA mouse model	Daily oral gavage with fisetin (20 mg/kg)	Vehicle	8 weeks	Benefit	- Fisetin has an anti-inflammatory effect and attenuates OA progression. - Fisetin-treated mice exhibited less cartilage destruction, reduced subchondral bone plate thickness, alleviated synovitis, and lower OARSI scores.
[133]	Collagenase-induced OA mouse model	- Intra-articular injection of human bone marrow-derived MSCs (2 × 10^4^ cells) - Intra-articular injection of the secretome from human bone marrow-derived MSCs (2 × 10^4^ cells)	Growth medium	1 and 3 weeks	Benefit	- Injection of MSC secretome, similarly to injection of MSCs, resulted in early pain reduction and had a protective effect on cartilage damage development. - No effects were observed regarding synovial inflammation, subchondral bone volume, or the presence of different macrophage subtypes.
[134]	Surgically-induced OA goat model	- Intra-articular injection of naïve human adipose-derived MSCs (0.6 × 10^7^) - Intra-articular injection of SOX-6, 9-transfected human adipose-derived MSCs in three doses (low-dose group: 0.18 × 10^7^ mid-dose group: 0.6 × 10^7^ high-dose group: 1.8 × 10^7^)	PBS	5 months	Benefit	- MSCs reduced OA progression in goats. - SOX-6, 9-transfected MSCs at a dose of 0.6 × 10^7^ best preserved articular cartilage and produced significantly better macroscopic and microscopic scores than negative controls in femoral and tibial articular surfaces.
[135]	Surgically-induced OA mouse model	- Intra-articular injection of exosomes from bone marrow-derived MSCs (BMSC-Exos) - Intra-articular injection of exosomes from curcumin-treated bone marrow-derived MSCs (CUR BMSC-Exos)	PBS	N/A	Benefit	- BMSC-Exos attenuated the progression of osteoarthritis. - CUR BMSC-Exos decreased the DNA methylation of miR-143 and miR-124 promoters. As a result, miR-143 and miR-124 were up-regulated to further inhibit the expression of their target genes, ROCK1 and NF-kB, which were closely related to the development of OA.
[136]	Ovariectomized bone loss rat model	Daily oral gavage with fisetin (5, 10, and 20 mg/kg)	Vehicle	16 weeks	Benefit	Fisetin improved bone density, bone mineral content, and biomechanical parameters.

## Data Availability

Not applicable.
